# Drug tolerability and reasons for discontinuation of seven biologics in 4466 treatment courses of rheumatoid arthritis—the ANSWER cohort study

**DOI:** 10.1186/s13075-019-1880-4

**Published:** 2019-04-11

**Authors:** Kosuke Ebina, Motomu Hashimoto, Wataru Yamamoto, Toru Hirano, Ryota Hara, Masaki Katayama, Akira Onishi, Koji Nagai, Yonsu Son, Hideki Amuro, Keiichi Yamamoto, Yuichi Maeda, Koichi Murata, Sadao Jinno, Tohru Takeuchi, Makoto Hirao, Atsushi Kumanogoh, Hideki Yoshikawa

**Affiliations:** 1 Department of Orthopaedic Surgery, Osaka University, Graduate School of Medicine, Osaka, Japan; 2 Department of Advanced Medicine for Rheumatic Diseases, Graduate School of Medicine, Kyoto University, Kyoto, Japan; 3 Department of Health Information Management, Kurashiki Sweet Hospital, Kurashiki, Japan; 4 Department of Respiratory Medicine and Clinical Immunology, Osaka University Graduate School of Medicine, Osaka, Japan; 5 The Center for Rheumatic Diseases, Nara Medical University, Nara, Japan; 6 Department of Rheumatology, Osaka Red Cross Hospital, Osaka, Japan; 7 Department of Rheumatology and Clinical Immunology, Kobe University Graduate School of Medicine, Kobe, Japan; 8 Department of Internal Medicine (IV), Osaka Medical College, Osaka, Japan; 9 First Department of Internal Medicine, Kansai Medical University, Osaka, Japan; 10 Department of Medical Informatics, Wakayama Medical University Hospital, Wakayama, Japan

**Keywords:** ANSWER cohort, Biological disease-modifying antirheumatic drugs, Discontinuation, Rheumatoid arthritis

## Abstract

**Background:**

The aim of this study is to evaluate the retention rates and reasons for discontinuation for seven biological disease-modifying antirheumatic drugs (bDMARDs) in a real-world setting of patients with rheumatoid arthritis (RA).

**Methods:**

This multi-center, retrospective study assessed 4466 treatment courses of 2494 patients with bDMARDs from 2009 to 2017 (females, 82.4%; baseline age, 57.4 years; disease duration 8.5 years; rheumatoid factor positivity 78.6%; Disease Activity Score in 28 joints using erythrocyte sedimentation rate, 4.3; concomitant prednisolone (PSL) 2.7 mg/day (43.1%) and methotrexate (MTX) 5.0 mg/week (61.8%); and 63.6% patients were bio-naïve). Treatment courses included tocilizumab (TCZ; *n* = 895), etanercept (ETN; *n* = 891), infliximab (IFX; *n* = 748), abatacept (ABT; *n* = 681), adalimumab (ADA; *n* = 558), golimumab (GLM; *n* = 464), and certolizumab pegol (CZP; *n* = 229). Drug retention rates and discontinuation reasons were estimated at 36 months using the Kaplan-Meier method and adjusted for potential confounders (age, sex, disease duration, concomitant PSL and MTX, and switched number of bDMARDs) using Cox proportional hazards modeling.

**Results:**

A total of 56.9% of treatment courses were stopped, with 25.8% stopping due to lack of effectiveness, 12.7% due to non-toxic reasons, 11.9% due to toxic adverse events, and 6.4% due to disease remission. Drug retention rates for each discontinuation reason were as follows: lack of effectiveness [from 65.5% (IFX) to 81.7% (TCZ); with significant differences between groups (Cox *P* < 0.001)], toxic adverse events [from 81.8% (IFX) to 94.0% (ABT), Cox *P* < 0.001], and remission [from 92.4% (ADA and IFX) to 97.7% (ETN), Cox *P* < 0.001]. Finally, overall retention rates excluding non-toxic reasons and remission for discontinuation ranged from 53.4% (IFX) to 75.5% (ABT) (Cox *P* < 0.001).

**Conclusions:**

TCZ showed the lowest discontinuation rate by lack of effectiveness, ABT showed the lowest discontinuation rate by toxic adverse events, ADA and IFX showed the highest discontinuation rate by remission, and ABT showed the highest overall retention rates (excluding non-toxic reasons and remission) among seven bDMARDs in the adjusted model.

## Introduction

Biological disease-modifying antirheumatic drugs (bDMARDs) have dramatically revolutionized the treatment of rheumatoid arthritis (RA). Tumor necrosis factor inhibitors (TNFi) were the first bDMARDs used for RA, and evidence has accumulated regarding the safety, effectiveness, and tolerability of adalimumab (ADA), etanercept (ETN), and infliximab (IFX) [1–5]. However, other TNFi such as golimumab (GLM) (2011) and certolizumab pegol (CZP) (2013) only recently received approval in Japan. The European League Against Rheumatism (EULAR) announced a 2016 recommendation regarding the management of RA with bDMARDs, in which CTLA4-Ig [abatacept (ABT)] and anti-interleukin (IL)-6 receptor antibody [tocilizumab (TCZ)] are considered as efficacious and safe as TNFi [6]. However, the clinician’s choice of bDMARD may depend on various factors (patients’ background characteristics such as age, comorbidities, use of conventional synthetic DMARDs (csDMARDs), previously administered bDMARDs, and economic burden), and reliable selection criteria for bDMARDs are still lacking.

The adaptive criterion of randomized controlled trials (RCTs) sometimes recruits patients who are different from those in real-world settings [7], and cohort-based observational studies have increasingly been used to investigate the performance of bDMARDs [1–4, 8–10]. Drug retention in observational studies is considered an index of safety, effectiveness, and tolerability [4, 11–13]. Treatment selection and discontinuation may be influenced by factors such as differences among attending physicians and patient characteristics in observational studies, although the national health insurance in our country and multicenter studies may help to decrease these possible bias (bDMARDs can be freely selected by attending physicians’ discretion in our country) [11–13].

We recently reported drug retention and reasons for discontinuation among seven biologics [14] and factors associated with the achievement of bDMARD-free remission [15] in our multicenter, retrospective RA cohort. However, these studies included a relatively small number of treatment courses (*n* = 1037; *n* = 181, respectively), and we added the patients’ number by consecutively collecting the data. The aim of this multicenter, retrospective study was to clarify the retention rates and reasons for discontinuation of seven biologics in the real-world setting of RA, with a larger number of treatment courses (*n* = 4466) compared with other previous cohort-based observational studies [1–3, 8–10].

## Materials and methods

### Patients

The Kansai Consortium for Well-being of Rheumatic Disease Patients (ANSWER) cohort is an observational multicenter registry of patients with RA in the Kansai district of Japan. Data from patients at seven institutes (Kyoto University, Osaka University, Osaka Medical College, Kansai Medical University, Kobe University, Nara Medical University, and Osaka Red Cross Hospital) were included. From 2009 to 2017, 4461 patients with RA were registered, and 52,654 serial disease activities were available from the database. Data from patients with RA treated using one of seven bDMARDs introduced between January 2009 and September 2017 (ABT, ADA, CZP, ETN, GLM, IFX, and TCZ; including both intravenous and subcutaneous agents, but excluding bio-similar agents) were retrospectively collected. In this study, patients who fulfilled the 1987 RA classification criteria of the American College of Rheumatology [16], with data on starting and discontinuation dates for bDMARDs, and reasons for discontinuation, were included. In addition, baseline demographic data such as age, sex, disease activity (Disease Activity Score in 28 joints using erythrocyte sedimentation rate [DAS28-ESR]), clinical disease activity index (CDAI), duration of RA, number of previously administered bDMARDs, concomitant doses of methotrexate (MTX) and prednisolone (PSL), rheumatoid factor (RF) and anti-cyclic citrullinated peptide antibody (ACPA) positivity, and Health Assessment Questionnaire [HAQ] disability index [DI] score were also collected.

Treatments were administered by the attending rheumatologists in accordance with guidelines of the Japan College of Rheumatology. Drug retention was retrospectively evaluated as the duration until definitive treatment interruption. Reasons for discontinuation were analyzed and classified into four major categories: (1) lack of effectiveness (including primary and secondary), (2) disease remission, (3) toxic adverse events (infection, skin or systemic reaction, and other toxic events, including hematologic, pulmonary, renal, cardiovascular complications, and malignancies, etc.), and (4) non-toxic reasons (patient preference, change in hospital, desire for pregnancy, etc.). Physicians were allowed to cite only one reason for discontinuation.

### Statistical analysis

Baseline characteristics were compared across the seven bDMARDs. The significance of differences was assessed using the Kruskal-Wallis nonparametric test for continuous variables and Pearson’s chi-square test for categorical variables. The survival curves of each biologic explained by specific causes were examined by the Kaplan-Meier method and compared statistically using a stratified log-rank test. The time to discontinuation of biologics was analyzed using multivariate Cox proportional hazards modeling [1]. The proportion of treatment retention rates explained by specific causes was analyzed at 36 months [14] and also adjusted by potential confounders that may influence drug discontinuation and the incidence of adverse events, as previously described (sex, baseline age, disease duration, concomitant treatment with MTX and PSL, and number of previously administered bDMARDs) [1, 8–10, 17]. Statistical analyses were performed using EZR (Saitama Medical Center, Jichi Medical University, Saitama, Japan), a graphical user interface for R (The R Foundation for Statistical Computing, Vienna, Austria) [18]. *P* < 0.05 was considered statistically significant.

## Results

### Baseline characteristics

The study population was selected from all patients with RA in the ANSWER cohort (*n* = 4461) who fulfilled the inclusion criteria (*n* = 2494; 4466 bDMARD treatment courses). Baseline demographic and clinical characteristics of patients are shown in Table 1. Overall at baseline, mean age was 57.4 years, 82.4% of participants were female, mean disease duration was 8.5 years, RF positivity was 78.6%, ACPA positivity was 82.4%, mean DAS28-ESR score was 4.3, CDAI was 16.7, and mean HAQ-DI score was 1.1. In addition, concomitant medications were PSL 2.7 mg/day (43.1%) and MTX 5.0 mg/week (61.8%). The bDMARD is being administered for the first agent in 63.6% of treatment courses, for the second agent in 22.4% of treatment courses, and for the third or latter agent in 14.0% of treatment courses.

**Table 1:** Clinical characteristics at initiation of each biologic agent

Variable	ABT (*n* = 681)	ADA (*n* = 558)	CZP (*n* = 229)	ETN (*n* = 891)	GLM (*n* = 464)	IFX (*n* = 748)	TCZ (*n* = 895)	*P* value
Age (years)	63.9 ± 13.0	55.5 ± 13.5	56.3 ± 16.3	55.7 ± 15.8	61.2 ± 14.7	52.8 ± 13.5	57.6 ± 14.2	< 0.001
Female sex (%)	81.2	81.7	87.7	85.0	87.3	78.0	80.8	< 0.001
BMI (kg/m^2^)	21.9 ± 3.7	22.3 ± 4.1	22.3 ± 3.3	21.9 ± 3.7	22.2 ± 3.5	22.3 ± 4.2	22.2 ± 3.9	0.81
Disease duration (years)	9.5 ± 10.3	7.9 ± 9.6	6.8 ± 8.9	9.1 ± 9.0	10.7 ± 10.7	7.4 ± 8.9	9.2 ± 9.2	< 0.001
RF positivity (%)	83.0	75.4	82.2	80.3	77.4	74.5	78.2	0.014
ACPA positivity (%)	84.6	77.4	85.4	84.2	78.9	82.8	82.9	0.036
DAS28-ESR	4.4 ± 1.3	4.1 ± 1.2	4.5 ± 1.5	4.3 ± 1.4	4.1 ± 1.3	4.4 ± 1.6	4.5 ± 1.4	< 0.001
CDAI	16.7 ± 9.8	14.0 ± 9.1	19.6 ± 12.3	15.9 ± 9.4	15.7 ± 10.8	18.6 ± 12.4	17.0 ± 10.1	0.0025
HAQ-DI	1.1 ± 0.8	0.7 ± 0.7	1.2 ± 0.8	0.9 ± 0.8	1.1 ± 0.8	1.1 ± 0.9	1.2 ± 0.8	< 0.001
PSL usage (%)	48.4	36.1	43.4	42.0	42.6	37.2	49.4	< 0.001
PSL dose (mg/day)	3.4 ± 6.9	2.2 ± 4.4	2.4 ± 3.7	2.5 ± 4.1	2.3 ± 3.5	2.2 ± 4.2	3.1 ± 5.3	0.011
MTX usage (%)	47.9	67.0	70.6	41.2	70.8	98.9	52.1	< 0.001
MTX dose (mg/week)	3.9 ± 4.6	6.0 ± 4.9	6.3 ± 4.8	3.3 ± 4.4	6.0 ± 4.7	8.2 ± 2.5	4.4 ± 4.8	< 0.001
1st bio (%)	59.2	69.9	59.0	72.4	45.5	89.4	43.4	< 0.001
2nd bio (%)	22.2	22.9	17.5	20.5	32.1	7.6	32.7	< 0.001
≥ 3rd bio (%)	18.6	7.2	23.5	7.1	22.4	3.0	23.9	< 0.001

Values represent mean ± standard deviation (SD), unless otherwise noted. Differences between drugs were assessed using the Kruskal-Wallis nonparametric test for continuous variables and Pearson’s chi-square test for categorical variables

*ABT* abatacept, *ADA* adalimumab, *CZP* certolizumab pegol, *ETN* etanercept, *GLM* golimumab, *IFX* infliximab, *TCZ* tocilizumab, *BMI* body mass index, *RF* rheumatoid factor, *ACPA* anti-cyclic citrullinated peptide antibody, *DAS28-ESR* Disease Activity Score in 28 joints using erythrocyte sedimentation rate, *CDAI* clinical disease activity index, *HAQ-DI* Health Assessment Questionnaire disability index, *PSL* prednisolone, *MTX* methotrexate, *bio* biologic agent

### Drug retention

Overall, 2540 treatment courses (56.9%) were stopped by 36 months. A total of 1154 treatment courses (25.8%) were stopped due to lack of effectiveness, 569 treatment courses (12.7%) due to non-toxic reasons, 532 treatment courses (11.9%) due to toxic reasons (161 treatment courses [3.6%] due to infection, 269 treatment courses [6.0%] due to other adverse events such as hematologic, pulmonary, renal, or cardiovascular complications or malignancy, and 102 treatment courses [2.3%] due to skin or systemic reaction), and 285 treatment courses (6.4%) due to remission.

### Reasons for discontinuation

**Fig. 1:**
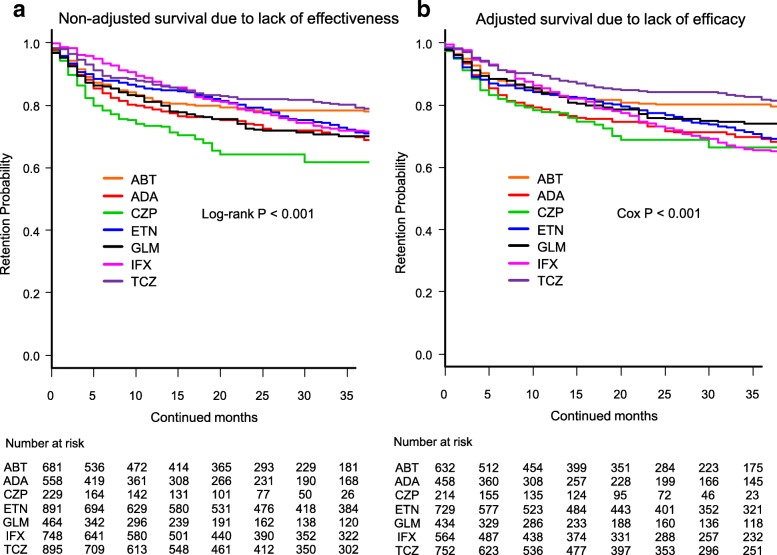
Drug survival rates due to lack of effectiveness in **a** non-adjusted cases and **b** adjusted cases. Adjusted confounders were baseline sex, age, and number of previously used bDMARDs. ABT = abatacept, ADA = adalimumab, CZP = certolizumab pegol, ETN = etanercept, GLM = golimumab, IFX = infliximab, TCZ = tocilizumab, bDMARDs = biological disease-modifying antirheumatic drugs

**Fig. 2:**
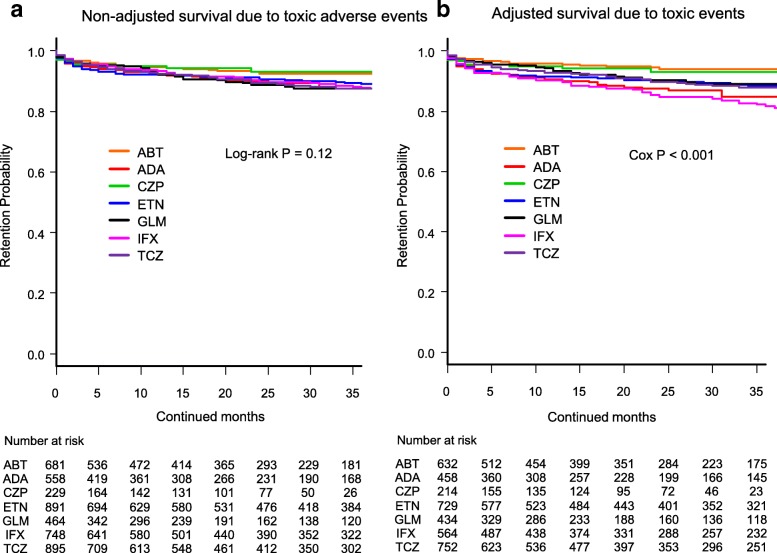
Drug survival rates due to toxic adverse events in **a** non-adjusted cases and **b** adjusted cases. Adjusted confounders were baseline sex, age, and number of previously used bDMARDs. ABT = abatacept, ADA = adalimumab, CZP = certolizumab pegol, ETN = etanercept, GLM = golimumab, IFX = infliximab, TCZ = tocilizumab, bDMARDs = biological disease-modifying antirheumatic drugs

**Fig. 3:**
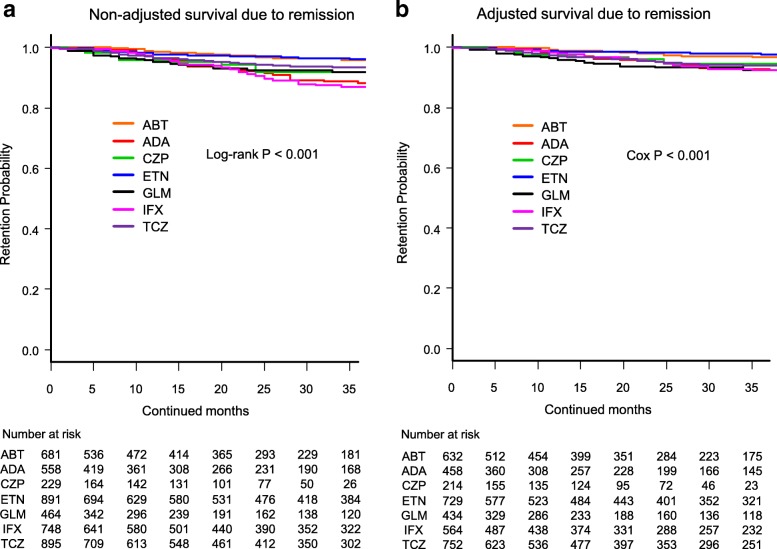
Drug survival rates due to remission in **a** non-adjusted cases and **b** adjusted cases. Adjusted confounders were baseline sex, age, and number of previously used bDMARDs. ABT = abatacept, ADA = adalimumab, CZP = certolizumab pegol, ETN = etanercept, GLM = golimumab, IFX = infliximab, TCZ = tocilizumab, bDMARDs = biological disease-modifying antirheumatic drugs

**Fig. 4:**
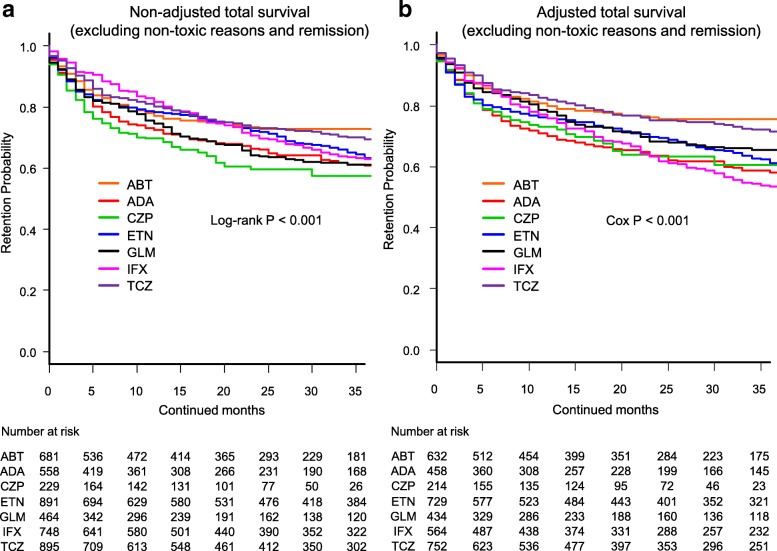
Overall drug survival rates (excluding non-toxic reasons and remission) in **a** non-adjusted cases and **b** adjusted cases. Adjusted confounders were baseline sex, age, and number of previously used bDMARDs. ABT = abatacept, ADA = adalimumab, CZP = certolizumab pegol, ETN = etanercept, GLM = golimumab, IFX = infliximab, TCZ = tocilizumab, bDMARDs = biological disease-modifying antirheumatic drugs

Cause-specific cumulative discontinuation rates were assessed using Kaplan-Meier estimates in both non-adjusted and adjusted models for cofounders using Cox proportional hazards regression modeling (Figs. 1, 2, 3, and 4). At 36 months, drug retention rates due to lack of effectiveness (Fig. 1) were as follows: (1) non-adjusted model: TCZ (79.4%), ABT (78.4%), IFX (71.8%), ETN (71.2%), GLM (70.2%), ADA (69.8%), and CZP (61.7%) (log-rank *P* < 0.001) (Fig. 1a), and (2) adjusted model: TCZ (81.7%), ABT (80.2%), GLM (74.0%), ETN (69.5%), ADA (69.1%), CZP (66.3%), and IFX (65.5%) (Cox *P* < 0.001) (Fig. 1b).

Drug retention rates due to all toxic adverse events (Fig. 2) were as follows: (1) non-adjusted model: CZP (93.1%), ABT (92.5%), ETN (89.2%), IFX (87.9%), ADA (87.5%), GLM (87.5%), and TCZ (87.5%) (log-rank *P* = 0.12) (Fig. 2a), and (2) adjusted model: ABT (94.0%), CZP (93.1%), GLM (89.1%), ETN (88.5%), TCZ (87.8%), ADA (84.7%), and IFX (81.8%) (Cox *P* < 0.001) (Fig. 2b).

Drug retention rates due to remission (Fig. 3) were as follows: (1) non-adjusted model: IFX (86.9%), ADA (88.1%), GLM (91.7%), CZP (91.9%), TCZ (93.1%), ABT (95.8%), and ETN (96.2%) (log-rank *P* < 0.001) (Fig. 3a), and (2) adjusted model: ADA (92.4%), IFX (92.4%), GLM (92.5%), TCZ (94.1%), CZP (94.5%), ABT (96.6%), and ETN (97.7%) (Cox *P* < 0.001) (Fig. 3b).

Total drug retention rates (excluding non-toxic reasons and remission) were analyzed using Kaplan-Meier estimates in both the non-adjusted model (Fig. 4a) and adjusted model using Cox proportional hazards regression modeling (Fig. 4b). At 36 months, drug retention rates were as follows: (1) non-adjusted model: ABT (72.7%), TCZ (69.4%), ETN (63.4%), IFX (63.1%), GLM (61.3%), ADA (60.9%), and CZP (57.4%) (log-rank *P* < 0.001), and (2) adjusted model: ABT (75.5%), TCZ (71.5%), GLM (65.6%), ETN (61.2%), CZP (60.7%), ADA (58.2%), and IFX (53.4%) (Cox *P* < 0.001).

Hazard ratios (HRs) and 95% confidence intervals (CI) for discontinuation due to each specific cause at 36 months were calculated using multivariate Cox proportional hazards modeling, adjusted for sex, baseline age, disease duration, concomitant treatment with MTX and PSL, and number of previously administered bDMARDs (Table 2). HRs for total discontinuation (excluding non-toxic reasons and remission) were significantly lower with ABT (HR = 0.56, 95%CI = 0.46–0.68, *P* < 0.001), TCZ (HR = 0.59, 95%CI = 0.49–0.71, *P* < 0.001), and GLM (HR = 0.71, 95%CI = 0.58–0.88, *P* = 0.002) compared with IFX, and significant differences were seen between the seven bDMARDs (*P* < 0.001). In terms of HRs for discontinuation due to lack of effectiveness, TCZ (HR = 0.54, 95%CI = 0.43–0.67, *P* < 0.001), ABT (HR = 0.65, 95%CI = 0.52–0.82, *P* < 0.001), and GLM (HR = 0.74, 95%CI = 0.57–0.95, *P* = 0.002) showed significantly lower rates compared with IFX. Differences were significant between the seven bDMARDs (*P* < 0.001).

**Table 2:** Causes of treatment discontinuation at 36 months (Cox proportional hazards model, adjusted analysis)

Variable	Reference	HR (95% CI)						
	IFX (*n* = 748)	ABT (*n* = 681)	ADA (*n* = 558)	CZP (*n* = 229)	ETN (*n* = 891)	GLM (*n* = 464)	TCZ (*n* = 895)	*P* value
Total discontinuation (excluding non-toxic reasons and remission)	1	0.56 (0.46–0.68)***	0.99 (0.82–1.20)	0.96 (0.74–1.23)	0.92 (0.78–1.08)	0.71 (0.58–0.88) **	0.59 (0.49–0.71)***	< 0.001
Lack of effectiveness	1	0.65 (0.52–0.82)***	1.03 (0.82–1.29)	1.16 (0.87–1.55)	0.97 (0.80–1.17)	0.74 (0.57–0.95) **	0.54 (0.43–0.67)***	< 0.001
All toxic adverse events	1	0.32 (0.22–0.49)***	0.83 (0.58–1.18)	0.43 (0.24–0.78)**	0.66 (0.47–0.93)*	0.57 (0.38–0.85)**	0.61 (0.43–0.85)**	< 0.001
Non-toxic reasons	1	0.92 (0.64–1.34)	0.98 (0.67–1.42)	0.40 (0.18–0.87)*	0.84 (0.60–1.18)	1.20 (0.83–1.77)	0.84 (0.60–1.19)	0.12
Remission	1	0.35 (0.20–0.60)***	0.98 (0.67–1.44)	0.80 (0.41–1.56)	0.40 (0.26–0.60)***	0.96 (0.60–1.56)	0.77 (0.52–1.14)	< 0.001

Differences between drugs were assessed using the Cox-P value.

*HR* hazard ratio; *95%CI* 95% confidence interval, *IFX* infliximab, *ABT* abatacept, *ADA* adalimumab, *CZP* certolizumab pegol, *ETN* etanercept, *GLM* golimumab, *TCZ* tocilizumab

*P < 0.05, **P < 0.01, ***P < 0.001

In terms of HRs for discontinuation due to all toxic adverse events, ABT (HR = 0.32, 95%CI = 0.22–0.49, *P* < 0.001), CZP (HR = 0.43, 95%CI = 0.24–0.78, *P* = 0.006), TCZ (HR = 0.61, 95%CI = 0.43–0.85, *P* = 0.004), and ETN (HR = 0.66, 95%CI = 0.47–0.93, *P* = 0.02) showed a significantly lower rate compared with IFX, and the difference was significant between the seven bDMARDs (*P* < 0.001).

No significant differences were observed in HRs for discontinuation due to non-toxic reasons between the seven bDMARDs (*P* = 0.12). On the other hand, IFX showed a higher HR for discontinuation due to remission compared with ABT (HR = 0.35, 95%CI = 0.20–0.60, *P* < 0.001) and ETN (HR = 0.40, 95%CI = 0.26–0.60 *P* < 0.001), and the difference was significant between the seven bDMARDs (P < 0.001).

In terms of other possible confounders, number of previously administered bDMARDs (HR = 1.25, 95%CI = 1.20–1.31, *P* < 0.001), concomitant PSL (HR = 1.25, 95%CI = 1.13–1.40, *P* < 0.001), male sex (HR = 1.23, 95%CI = 1.07–1.41, *P* = 0.004), and higher age (HR = 1.004, 95%CI = 1.001–1.008, *P* = 0.02) at baseline showed negative effects on total drug retention rates (excluding non-toxic reasons and remission).

## Discussion

This study was designed to evaluate the retention rates and reasons for discontinuation for seven bDMARDs in a real-world setting of patients with RA, with relatively a larger number of treatment courses compared to other previous reports.

With respect to the differences between TNFi and non-TNFi, we have previously reported that TCZ showed greater effectiveness and a higher retention rate compared with ADA and IFX [19], and both ABT and TCZ showed lower rate of lack of effectiveness  and a higher retention rate compared with other TNFi [14]. In addition, in patients in whom TNFi failed, both ABT and TCZ showed good-or-moderate EULAR response (ABT 77%, TCZ 84%) at 48 weeks in DANBIO registry [20]. Another report also showed that in patients with first TNFi failure, switching to a non-TNFi bDMARD was associated with higher retention rates compared to switching to a second-TNFi after adjustment for propensity scores [8]. Collectively, ABT and TCZ may exhibit higher retention rates compared with other TNFi in both bio-naïve and bio-switched patients. This phenomenon may be partially due to small dose and ratio of concomitant MTX in this study, which may affect TNFi effectiveness more stronger than that of non-TNFi.

In terms of toxic adverse events, a recent report demonstrated that among patients with RA using biologic agents, the risk for infection leading to hospitalization was the lowest with ABT compared with other bDMARDs [21]. In addition, the incidence of serious infections across bDMARDs in patients with RA was not higher with CZP compared with other bDMARDs [22]. Another recent report showed that the risk for toxic adverse events such as lupus-like events and vasculitis-like events in TNFi-treated patients with RA tended to be the lowest with CZP compared with other bDMARDs [23]. Taken together, ABT and CZP may exhibit lower toxic adverse events compared with other bDMARDs.

In terms of stopping bDMARDs due to remission, previous reports have demonstrated that IFX and ADA seem to have better potential to be stopped due to remission compared with CZP or ETN, as was shown in the BeSt, HIT HARD, and OPTIMA studies in patients with early RA, and in the RRR and HONOR studies in patients with established RA [24–31]. This may be partially explained by a previous report demonstrating that monoclonal anti-TNF antibodies (ADA and IFX) induced stronger complement-dependent cytotoxicity and apoptosis in transmembrane TNF alpha-expressing cells compared to ETN and rituximab in vitro [32]. This phenomenon may be favorable in obtaining deep clinical remission, although these previous reports may influence individual physician decisions regarding discontinuation in this study. Thus, we conducted a study to investigate the maintenance of bDMARD-free remission between these agents [15]. From our results, TNF monoclonal antibodies (IFX, ADA, and GLM) or ABT were more advantageous for achieving sustained bDMARD-free remission compared with soluble TNF receptor (ETN) or Fab fragments against TNF fused with polyethylene glycol (CZP) or IL-6 receptor antibody (TCZ). Taken together, TNF monoclonal antibodies (IFX, ADA, and GLM) may have some advantages in both achieving and maintaining bDMARD-free remission compared with other bDMARDs.

Factors affecting bDMARD retention and response other than differences in bDMARDs have been reported. Concomitant PSL [3], high DAS28 or HAQ [3, 9, 33], absence or low dose of combined MTX [3, 9], and the number of previously used bDMARDs [9] were negative predictors, which is consistent with the results of our previous study [14]. However, selection of bDMARDs may depend on these background factors in routine care, and indeed, significant differences were observed in these backgrounds between bDMARDs groups in the present study. Adjusting for all these factors may not always reflect what happens in routine care; therefore, we conducted both non-adjusted model and adjusted model by sex, age, disease duration, concomitant treatment with MTX and PSL, and number of previously treated bDMARDs. Finally, compared with our previous study [14], GLM showed lower rate of lack of effectiveness and higher rate of discontinuation due to remission, and CZP and TCZ showed lower rate of toxic event in adjusted model.

Regarding the efficacy of low-dose MTX in Japanese populations compared with western populations, intraerythrocyte MTX-polyglutamate (MTX-PG) concentrations, which have been suggested to be a useful biomarker of efficacy, reached 94 nmol/L with 10.3 mg/week of MTX in Japanese, compared to 65 nmol/L with 13.4 mg/week of MTX in the USA [34]. As a result, a relatively low dose of MTX may exhibit positive effects on bDMARD retention in Japanese populations compared with western populations.

Some limitations to this study need to be considered. First, the judgment and reasons for discontinuation (such as lack of effectiveness or remission) depended on the decisions of each physician, without standardized criteria. Second, the backgrounds of patients differed between the agents, which may affect the results even adjusted by potent cofounders. Third, the minor dose changes of bDMARDs, MTX, and PSL could not be monitored. Fourth, the difference of intravenous and subcutaneous bDMARDs and the presence of other csDMARDs could not be determined. Fifth, we could not fully adjust the data of comorbidities, disease activity, and HAQ before 2011, which may affect the retention rates. Sixth, CZP was licensed most recently (2013) among seven bDMARDs in our country, which may lead to smaller number of prescription that may affect the results.

However, the strengths of this study were relatively a large number of treatment courses of seven bDMARDs, and that treatment choice and discontinuation judgments were based on a real-world setting.

## Conclusions

TCZ showed the lowest discontinuation rate by lack of effectiveness, ABT showed the lowest discontinuation rate by toxic adverse events, ADA and IFX showed the highest discontinuation rate by remission, and ABT showed the highest overall retention rate (excluding non-toxic reasons and remission) among seven bDMARDs in adjusted model.

## References

[1] Du Pan SM, Dehler S, Ciurea A, Ziswiler HR, Gabay C, Finckh A (2009). Comparison of drug retention rates and causes of drug discontinuation between anti-tumor necrosis factor agents in rheumatoid arthritis. Arthritis Rheum.

[2] Favalli EG, Pregnolato F, Biggioggero M, Becciolini A, Penatti AE, Marchesoni A (2016). Twelve-year retention rate of first-line tumor necrosis factor inhibitors in rheumatoid arthritis: real-life data from a local registry. Arthritis Care Res (Hoboken).

[3] Hetland ML, Christensen IJ, Tarp U, Dreyer L, Hansen A, Hansen IT (2010). Direct comparison of treatment responses, remission rates, and drug adherence in patients with rheumatoid arthritis treated with adalimumab, etanercept, or infliximab: results from eight years of surveillance of clinical practice in the nationwide Danish DANBIO registry. Arthritis Rheum.

[4] Neovius M, Arkema EV, Olsson H, Eriksson JK, Kristensen LE, Simard JF (2015). Drug survival on TNF inhibitors in patients with rheumatoid arthritis comparison of adalimumab, etanercept and infliximab. Ann Rheum Dis.

[5] Souto A, Maneiro JR, Gomez-Reino JJ (2016). Rate of discontinuation and drug survival of biologic therapies in rheumatoid arthritis: a systematic review and meta-analysis of drug registries and health care databases. Rheumatology (Oxford).

[6] Smolen JS, Landewe R, Bijlsma J, Burmester G, Chatzidionysiou K, Dougados M (2017). EULAR recommendations for the management of rheumatoid arthritis with synthetic and biological disease-modifying antirheumatic drugs: 2016 update. Ann Rheum Dis.

[7] Wolfe F, Michaud K, Dewitt EM (2004). Why results of clinical trials and observational studies of antitumour necrosis factor (anti-TNF) therapy differ: methodological and interpretive issues. Ann Rheum Dis.

[8] Favalli EG, Biggioggero M, Marchesoni A, Meroni PL (2014). Survival on treatment with second-line biologic therapy: a cohort study comparing cycling and swap strategies. Rheumatology (Oxford).

[9] Gabay C, Riek M, Scherer A, Finckh A (2015). Effectiveness of biologic DMARDs in monotherapy versus in combination with synthetic DMARDs in rheumatoid arthritis: data from the Swiss Clinical Quality Management Registry. Rheumatology (Oxford).

[10] Jorgensen TS, Kristensen LE, Christensen R, Bliddal H, Lorenzen T, Hansen MS (2015). Effectiveness and drug adherence of biologic monotherapy in routine care of patients with rheumatoid arthritis: a cohort study of patients registered in the Danish biologics registry. Rheumatology (Oxford).

[11] Hjardem E, Hetland ML, Ostergaard M, Krogh NS, Kvien TK (2005). Prescription practice of biological drugs in rheumatoid arthritis during the first 3 years of post-marketing use in Denmark and Norway: criteria are becoming less stringent. Ann Rheum Dis.

[12] Hyrich KL, Watson KD, Lunt M, Symmons DP (2011). Changes in disease characteristics and response rates among patients in the United Kingdom starting anti-tumour necrosis factor therapy for rheumatoid arthritis between 2001 and 2008. Rheumatology (Oxford).

[13] Simard JF, Arkema EV, Sundstrom A, Geborek P, Saxne T, Baecklund E (2011). Ten years with biologics: to whom do data on effectiveness and safety apply?. Rheumatology (Oxford).

[14] Ebina K, Hashimoto M, Yamamoto W, Ohnishi A, Kabata D, Hirano T (2018). Drug retention and discontinuation reasons between seven biologics in patients with rheumatoid arthritis -the ANSWER cohort study. PLoS One.

[15] Hashimoto M, Furu M, Yamamoto W, Fujimura T, Hara R, Katayama M (2018). Factors associated with the achievement of biological disease-modifying antirheumatic drug-free remission in rheumatoid arthritis: the ANSWER cohort study. Arthritis Res Ther.

[16] Arnett FC, Edworthy SM, Bloch DA, McShane DJ, Fries JF, Cooper NS (1988). The American rheumatism association 1987 revised criteria for the classification of rheumatoid arthritis. Arthritis Rheum.

[17] Greenberg JD, Reed G, Decktor D, Harrold L, Furst D, Gibofsky A (2012). A comparative effectiveness study of adalimumab, etanercept and infliximab in biologically naive and switched rheumatoid arthritis patients: results from the US CORRONA registry. Ann Rheum Dis.

[18] Kanda Y (2013). Investigation of the freely available easy-to-use software 'EZR' for medical statistics. Bone Marrow Transplant.

[19] Hishitani Y, Ogata A, Shima Y, Hirano T, Ebina K, Kunugiza Y (2013). Retention of tocilizumab and anti-tumour necrosis factor drugs in the treatment of rheumatoid arthritis. Scand J Rheumatol.

[20] Leffers HC, Ostergaard M, Glintborg B, Krogh NS, Foged H, Tarp U (2011). Efficacy of abatacept and tocilizumab in patients with rheumatoid arthritis treated in clinical practice: results from the nationwide Danish DANBIO registry. Ann Rheum Dis.

[21] Yun H, Xie F, Delzell E, Levitan EB, Chen L, Lewis JD (2016). Comparative risk of hospitalized infection associated with biologic agents in rheumatoid arthritis patients enrolled in Medicare. Arthritis Rheumatol.

[22] Rutherford AI, Subesinghe S, Hyrich KL, Galloway JB (2018). Serious infection across biologic-treated patients with rheumatoid arthritis: results from the British Society for Rheumatology Biologics Register for Rheumatoid Arthritis. Ann Rheum Dis.

[23] Jani M, Dixon WG, Kersley-Fleet L, Bruce IN, Chinoy H, Barton A (2017). Drug-specific risk and characteristics of lupus and vasculitis-like events in patients with rheumatoid arthritis treated with TNFi: results from BSRBR-RA. RMD Open.

[24] Detert J, Bastian H, Listing J, Weiss A, Wassenberg S, Liebhaber A (2013). Induction therapy with adalimumab plus methotrexate for 24 weeks followed by methotrexate monotherapy up to week 48 versus methotrexate therapy alone for DMARD-naive patients with early rheumatoid arthritis: HIT HARD, an investigator-initiated study. Ann Rheum Dis.

[25] Goekoop-Ruiterman YP, de Vries-Bouwstra JK, Allaart CF, van Zeben D, Kerstens PJ, Hazes JM (2005). Clinical and radiographic outcomes of four different treatment strategies in patients with early rheumatoid arthritis (the BeSt study): a randomized, controlled trial. Arthritis Rheum.

[26] Hirata S, Saito K, Kubo S, Fukuyo S, Mizuno Y, Iwata S (2013). Discontinuation of adalimumab after attaining disease activity score 28-erythrocyte sedimentation rate remission in patients with rheumatoid arthritis (HONOR study): an observational study. Arthritis Res Ther..

[27] Kavanaugh A, Fleischmann RM, Emery P, Kupper H, Redden L, Guerette B (2013). Clinical, functional and radiographic consequences of achieving stable low disease activity and remission with adalimumab plus methotrexate or methotrexate alone in early rheumatoid arthritis: 26-week results from the randomised, controlled OPTIMA study. Ann Rheum Dis.

[28] Smolen JS, Emery P, Ferraccioli GF, Samborski W, Berenbaum F, Davies OR (2015). Certolizumab pegol in rheumatoid arthritis patients with low to moderate activity: the CERTAIN double-blind, randomised, placebo-controlled trial. Ann Rheum Dis.

[29] Smolen JS, Nash P, Durez P, Hall S, Ilivanova E, Irazoque-Palazuelos F (2013). Maintenance, reduction, or withdrawal of etanercept after treatment with etanercept and methotrexate in patients with moderate rheumatoid arthritis (PRESERVE): a randomised controlled trial. Lancet..

[30] Tanaka Y, Hirata S, Saleem B, Emery P (2013). Discontinuation of biologics in patients with rheumatoid arthritis. Clin Exp Rheumatol.

[31] Tanaka Y, Takeuchi T, Mimori T, Saito K, Nawata M, Kameda H (2010). Discontinuation of infliximab after attaining low disease activity in patients with rheumatoid arthritis: RRR (remission induction by Remicade in RA) study. Ann Rheum Dis.

[32] Mitoma H, Horiuchi T, Tsukamoto H, Tamimoto Y, Kimoto Y, Uchino A (2008). Mechanisms for cytotoxic effects of anti-tumor necrosis factor agents on transmembrane tumor necrosis factor alpha-expressing cells: comparison among infliximab, etanercept, and adalimumab. Arthritis Rheum.

[33] Forsblad-d'Elia H, Bengtsson K, Kristensen LE, Jacobsson LT (2015). Drug adherence, response and predictors thereof for tocilizumab in patients with rheumatoid arthritis: results from the Swedish biologics register. Rheumatology (Oxford).

[34] Takahashi C, Kaneko Y, Okano Y, Taguchi H, Oshima H, Izumi K (2017). Association of erythrocyte methotrexate-polyglutamate levels with the efficacy and hepatotoxicity of methotrexate in patients with rheumatoid arthritis: a 76-week prospective study. RMD Open..

