# Correction to: Drug tolerability and reasons for discontinuation of seven biologics in 4466 treatment courses of rheumatoid arthritis—the ANSWER cohort study

**DOI:** 10.1186/s13075-019-1897-8

**Published:** 2019-05-06

**Authors:** Kosuke Ebina, Motomu Hashimoto, Wataru Yamamoto, Toru Hirano, Ryota Hara, Masaki Katayama, Akira Onishi, Koji Nagai, Yonsu Son, Hideki Amuro, Keiichi Yamamoto, Yuichi Maeda, Koichi Murata, Sadao Jinno, Tohru Takeuchi, Makoto Hirao, Atsushi Kumanogoh, Hideki Yoshikawa

**Affiliations:** 1 Department of Orthopaedic Surgery, Osaka University, Graduate School of Medicine, Osaka, Japan; 2 Department of Advanced Medicine for Rheumatic Diseases, Graduate School of Medicine, Kyoto University, Kyoto, Japan; 3 Department of Health Information Management, Kurashiki Sweet Hospital, Kurashiki, Japan; 4 Department of Respiratory Medicine and Clinical Immunology, Osaka University Graduate School of Medicine, Osaka, Japan; 5 The Center for Rheumatic Diseases, Nara Medical University, Nara, Japan; 6 Department of Rheumatology, Osaka Red Cross Hospital, Osaka, Japan; 7 Department of Rheumatology and Clinical Immunology, Kobe University Graduate School of Medicine, Kobe, Japan; 8 Department of Internal Medicine (IV), Osaka Medical College, Osaka, Japan; 9 First Department of Internal Medicine, Kansai Medical University, Osaka, Japan; 10 Department of Medical Informatics, Wakayama Medical University Hospital, Wakayama, Japan

**Correction to: Arthritis Res Ther**

**https://doi.org/10.1186/s13075-019-1880-4**

Following publication of the original article [1], the authors noticed that two corrections were not implemented during the production process. The original article [1] has been corrected.

In Fig. 1b, the heading should read: Adjusted survival due to lack of effectiveness

In Fig. 2b, the heading should read: Adjusted survival due to toxic adverse events

In addition, the authors would like to update the legends of Figure 1, 2, 3 and 4 to the following:

Fig. 1 Drug survival rates due to lack of effectiveness in **a** non-adjusted cases and **b** adjusted cases. Adjusted confounders were baseline sex, age, disease duration, concomitant prednisolone and methotrexate, and number of previously used bDMARDs. ABT = abatacept, ADA = adalimumab, CZP = certolizumab pegol, ETN = etanercept, GLM = golimumab, IFX = infliximab, TCZ = tocilizumab, bDMARDs = biological disease-modifying antirheumatic drugs

Fig. 2 Drug survival rates due to toxic adverse events in **a** non-adjusted cases and **b** adjusted cases. Adjusted confounders were baseline sex, age, disease duration, concomitant prednisolone and methotrexate, and number of previously used bDMARDs. ABT = abatacept, ADA = adalimumab, CZP = certolizumab pegol, ETN = etanercept, GLM = golimumab, IFX = infliximab, TCZ = tocilizumab, bDMARDs = biological disease-modifying antirheumatic drugs

Fig. 3 Drug survival rates due to remission in **a** non-adjusted cases and **b** adjusted cases. Adjusted confounders were baseline sex, age, disease duration, concomitant prednisolone and methotrexate, and number of previously used bDMARDs. ABT = abatacept, ADA = adalimumab, CZP = certolizumab pegol, ETN = etanercept, GLM = golimumab, IFX = infliximab, TCZ = tocilizumab, bDMARDs = biological disease-modifying antirheumatic drugs

Fig. 4 Overall drug survival rates (excluding non-toxic reasons and remission) in **a** non-adjusted cases and **b** adjusted cases. Adjusted confounders were baseline sex, age, disease duration, concomitant prednisolone and methotrexate, and number of previously used bDMARDs. ABT = abatacept, ADA = adalimumab, CZP = certolizumab pegol, ETN = etanercept, GLM = golimumab, IFX = infliximab, TCZ = tocilizumab, bDMARDs = biological disease-modifying antirheumatic drugs

The publishers apologise for the errors. The corrected figures are given below:

**Fig. 1:**
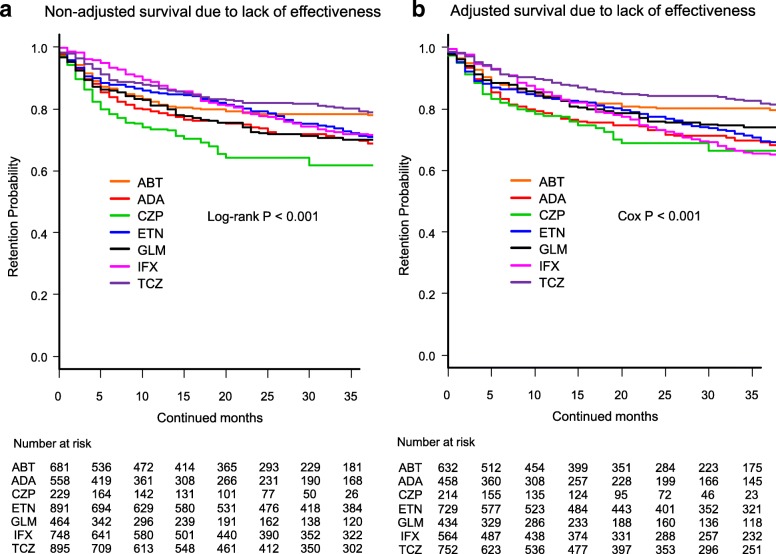
Drug survival rates due to lack of effectiveness in **a** non-adjusted cases and **b** adjusted cases. Adjusted confounders were baseline sex, age, disease duration, concomitant prednisolone and methotrexate, and number of previously used bDMARDs. ABT = abatacept, ADA = adalimumab, CZP = certolizumab pegol, ETN = etanercept, GLM = golimumab, IFX = infliximab, TCZ = tocilizumab, bDMARDs = biological disease-modifying antirheumatic drugs

**Fig. 2:**
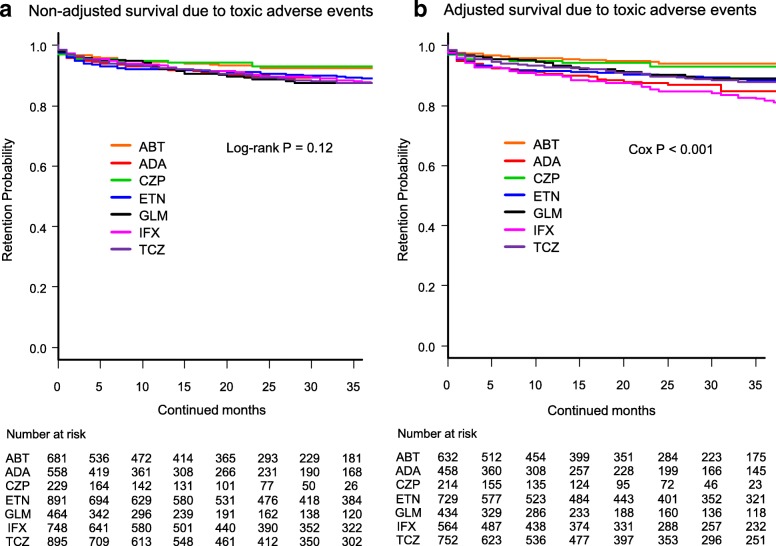
Drug survival rates due to toxic adverse events in **a** non-adjusted cases and **b** adjusted cases. Adjusted confounders were baseline sex, age, disease duration, concomitant prednisolone and methotrexate, and number of previously used bDMARDs. ABT = abatacept, ADA = adalimumab, CZP = certolizumab pegol, ETN = etanercept, GLM = golimumab, IFX = infliximab, TCZ = tocilizumab, bDMARDs = biological disease-modifying antirheumatic drugs
